# Supplementary figures and images for: Environmental exposure to metal mixtures and linear growth in healthy Ugandan children
Source: PLoS One. 2020 May 15;15(5):e0233108. doi: 10.1371/journal.pone.0233108 (PMC7228047; doi:10.1371/journal.pone.0233108)

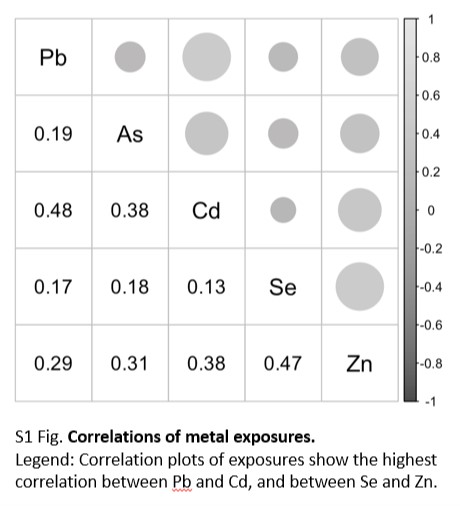

Supplement: S1 Fig — Legend: Correlation plots of metals exposures show the highest correlation between Pb and Cd, and between Se and Zn. (JPG) [file pone.0233108.s001.jpg]

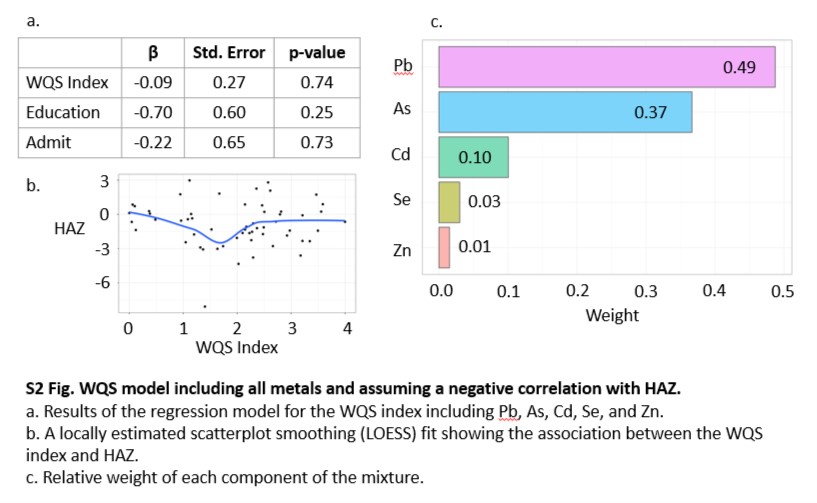

Supplement: S2 Fig — a. Results of the regression model for the WQS index of the metals (Pb, As, Cd, Se, Zn). b. A locally estimated scatterplot smoothing (LOESS) fit showing the association between the WQS index and HAZ. c. Relative weight of each metal in the mixture. (JPG) [file pone.0233108.s002.jpg]

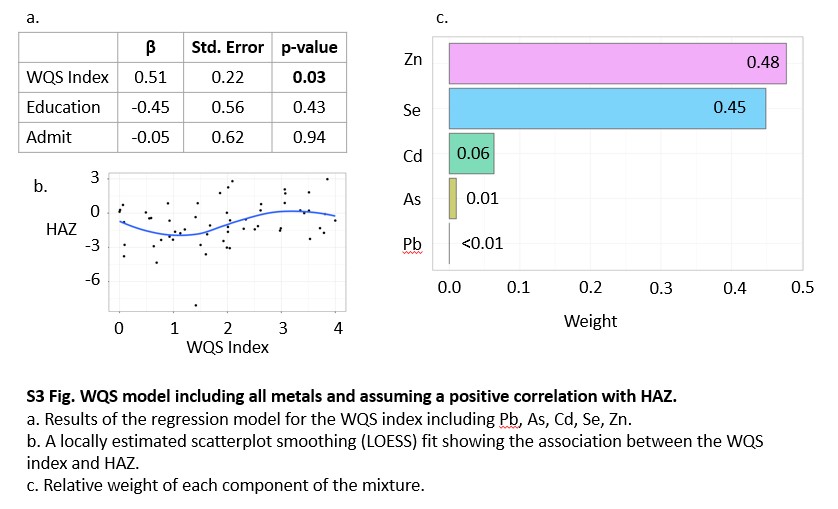

Supplement: S3 Fig — a. Results of the regression model for the WQS index of the metals (Pb, As, Cd, Se, Zn). b. A locally estimated scatterplot smoothing (LOESS) fit showing the association between the WQS index and HAZ. c. Relative weight of each metal in the mixture. (JPG) [file pone.0233108.s003.jpg]
