# Supplementary material for: Environmental exposure to metal mixtures and linear growth in healthy Ugandan children
Source: PLoS One. 2020 May 15;15(5):e0233108. doi: 10.1371/journal.pone.0233108 (PMC7228047; doi:10.1371/journal.pone.0233108)
Supplement: S1 Table — Caption: Unadjusted means of metal exposures for the study population (n = 97), and for the stunted (n = 22), and not stunted (n = 75) groups. (DOCX) [file pone.0233108.s004.docx]

**S1 Table. Mean exposure distributions**

|  | **All** | **Stunted** | **Not Stunted** |
| --- | --- | --- | --- |
| **Heavy metal** | **Mean (SD)** | **Mean (SD)** | **Mean (SD)** |
| **Pb (μg/dL)** | 6.24 (2.59) | 6.67 (2.19) | 6.11 (2.69) |
| **As (μg/L)** | 0.26 (0.16) | 0.29 (0.16) | 0.26 (0.16) |
| **Cd (μg/L)** | 0.09 (0.07) | 0.09 (0.05) | 0.09 (0.07) |
| **Se (μg/dL)** | 12.92 (2.91) | 11.86 (2.66) | 13.23 (2.92) |
| **Zn (mg/L)** | 3.72 (1.03) | 3.58 (0.779) | 3.76 (1.09) |
| Note: Pb, lead; As, arsenic; Cd, cadmium; Se, selenium; Zn, zinc; SD, standard deviation | | | |

Caption: Means of unadjusted measures trace element exposures for the study population (n=97), and for the stunted (n=22), and not stunted (n=75) groups.
