# Supplementary material for: Environmental exposure to metal mixtures and linear growth in healthy Ugandan children
Source: PLoS One. 2020 May 15;15(5):e0233108. doi: 10.1371/journal.pone.0233108 (PMC7228047; doi:10.1371/journal.pone.0233108)
Supplement: Attachment — Submitted filename: Response letter.docx [file pone.0233108.s006.docx]

January 17, 2020

Dear Editor and Reviewers,

Thank you for your thoughtful comments and suggestions on this paper. I have revised the paper according to your suggestions and think that the resulting paper is both stronger and clearer. I appreciate your time and contributions to this publication.

Sincerely,
Emily Moody

Reviewer #1:

To study whether toxic metals and essential nutrients were associated with growth of children (using height-for-age Z-score, HAZ). There were 97 children enrolled, aged from 6 to 59 months, and their whole blood samples were analyzed for arsenic (As), cadmium (Cd), lead (Pb), selenium (Se), and zinc (Zn) by ICP-MS at the Senator Frank R. Lautenberg Environmental Health Sciences Laboratory at the Icahn School of Medicine, Mount Sinai, NY. The result showed that lower Se and higher Pb blood levels were both associated with lower HAZ.

Abstract line 42, “Lower selenium and higher blood level were both associated with lower HAZ.” What were higher blood levels?

Were all the concentrations of metals in blood analyzed whole blood sample? I know Pb was analyzed with whole blood sample, however, for the other elements, especially, Se and Zn, the standard analysis method are serum. Please clarify this point and provide the method of preparing sample (pre-treatment).

Response: Yes, the analysis method included a single sample preparation from whole blood. Further information on sample preparation has been included, and references from previous publications employing this method. In addition, the inability to comment on Se or Zn deficiency with this sample source was commented on in the limitations section as well as the results sections. To limit the risk to the young children who participated we chose to take a single blood sample to minimize the amount of blood drawn, so we did not have enough to fractionate and analyze whole blood and serum samples.

Updated manuscript text: Page 6, Line 120-131: “Upon collection whole blood samples were refrigerated at 4^o^C and shipped to New York, USA for analysis. Whole blood samples (1 ml) were acid digested using concentrated nitric acid (2-ml) and hydrogen peroxide (1 ml) at room temperature for 48 hours prior dilution to 10-ml with deionized water and were analyzed using an Agilent 8800 ICP Triple Quad (ICP-QQQ) (Agilent technologies, Inc., Delaware, USA) in MS/MS mode with appropriate cell gases to eliminate molecular ion interferences. Using a method previously described, samples were analyzed for heavy metals including antimony, arsenic, barium, cadmium, cesium, chromium, cobalt, copper, lead, manganese, nickel, selenium, zinc using external calibration with appropriate internal standards (yttrium, indium, tellurium and lutetium) at the Senator Frank R. Lautenberg Environmental Health Sciences Laboratory at the Icahn School of Medicine, Mount Sinai, NY[CHITRA REFS PMID: 30849576, 31357156].”

Page 14, Line 302-303: “we were unable to determine Se deficiency using whole blood Se”

Page 15, Line 316-317: “whole blood Zn cannot be used to diagnose deficiency so we were unable to determine rates of Zn deficiency in this study.”

Page 17, Lines 353-354: “Finally, our measure of metals in whole blood precludes us from determining Zn or Se deficiency in this population of children.”

Table 2, please provide the mean and SD as well as median and IQR. Moreover, I suggest to divide into “Stunting” and “Non-stunting” groups, then using independent T test or nonparametric method to test the difference between these 2 groups.

Response: The mean and SD have been added to the table. Another column for the p-value from a wilcoxan rank sum test was added (stunted children vs non-stunted children) showing no significant differences between the stunted and non-stunted groups for any of the heavy metal concentrations.

This is an interesting manuscript. Thanks for giving me the chance to review…

Reviewer #2:

The manuscript associates the levels of Pb, Cd, As, Zn and Se with stunting in Ugandan children aged 6 to 60 months. Particularly, a negative association between the height-for-age score (HAZ) and Pb in blood and a positive association between HAZ and Se in blood. A new approach introducing a weighted quantile sum (WQS) statistics was applied in the attempt to evaluate the impact of multiple metals in blood on the levels of height for age score.

Some weaknesses are present in the manuscript. Particularly:

1-the living environment and the social status of study children were not included in the analysis, so these potential confounding factors were not taken into account in studying the association between HAZ and levels of metals in blood;
2-the score HAZ is built taking into account the different countries or it was built with US data and adapted to Ugandan children? Could you explain something more about HAZ?
3- the levels of Zn in blood reported in the study are much higher than those expected based on reference values for the general population. How can you explain this?
All this considering, I think that additional information should be included in the paper and that results of the study are only suggestive of a role played by Pb exposure on stunting, so that a major caution should be used in drawing conclusion about the detrimental health effect due to heavy metals.

Suggested revisions
**Methods**
Add information about the year of the field study;

Updated manuscript text: Page 6, Line 99 “In May 2016, “

Add the list of metals measured in the original study;

Updated manuscript text: Page 7, Lines 119-121 “…samples were analyzed for antimony, arsenic, barium, cadmium, cesium, chromium, cobalt, copper, lead, manganese, nickel, selenium, zinc were measured by LC-tandem mass spectrometry in the Senator Frank R. Lautenberg Environmental Health Sciences Laboratory at the Icahn School of Medicine, Mount Sinai, NY.

Add information about the living environment and the social status of the study children and explain their effect of the association between HAZ and metals in blood.

Response: The neighborhood where the children live, and from which they were recruited, is described in detail in the referenced publication.

Updated Manuscript text: Page 6, Lines 96-98 “The community and recruitment procedures are described in detail elsewhere (1). In short, all children were from the same low-lying community of makeshift temporary structures with poor drainage and garbage management.”

Add information about the HAZ score. Is this country specific? In the case this was calculated for US children, do the author have confidence about its applicability in Ugandan children?

Response: We calculated HAZ scores using Epi info version 3.5.1 These standards were developed form normal formula-fed children in the USA. When applied to this population in Uganda, there may be overestimation of estimates of poor growth in the <1 yo children because breastfed babies grow more slowly than formula fed babies. This has been added to the discussion of limitations of the study.

Updated manuscript text: Page 16, Lines 310-313 “We used Epi info version 3.5.1 to derive HAZ scores, which is based upon a sample of formula-fed babies in the USA in 1978, which could overestimate undernutrition in our sample since the rate of growth in formula-fed infants is greater than breast-fed in the first year of life.”

The authors describe WQS as a statistical tool for high-dimensional dataset; is the dataset used in this study big enough for the use with WQS?

Response: WQS is a statistical tool that can handle from small to high dimensional datasets. We have previously implemented WQS using a small dataset and found good performance of the WQS under those conditions (2). A WQS extension for high-dimension data is available when needed (3). This study did have a small sample size. To overcome the challenges of the smaller sample size, we use 40% of the observations to train the model and 60% of the observations to validate the model. We take confidence that the model performance is satisfactory from the example of another small dataset (N=200) that had a much higher-dimension dataset with a ratio of 5:1 of variables to observations (4). Our data set had a variable: observation ratio of 19.5:1.

**Results**
A R2 = 0.088 is very small; so the model multiple regression model is only marginally explaining the HAZ score.

Response: In many environmental studies, the R^2^ are generally small. We expect that had we been able to account for nutritional factors we would have been able to much better account for HAZ variation in the children. However, this R^2^ is very small which may be due to other conditions we are not aware of. To reduce confusion, we have removed this sentence from the manuscript.

The figures of the WQS regressions are not very clear. What is the meaning of the graph with the curve? Where is the HAZ score on the graph?

Response: Please excuse my oversight that the y-axis label for the graph with the curve was incorrectly labeled. It should say HAZ (our outcome) and this has been changed in all figures. The curve is a LOESS (locally estimated scatterplot smoothing) fit which represents the association between the WQS index and the outcome variable. The outcome variable is adjusted for all covariates, and a summary of each variables’ relative weight within that index.

Moreover, maybe the graphical representation of this WQS model could be limited to Fig 3 and Fig 4.

Response: This is reasonable. The results from the WQS analyses divided by direction of effect are the main findings for this paper, so the WQS figures for those findings remain as part of the main text. The secondary findings including all 5 metals in the WQS analyses have been moved to supporting information.

**Discussion**
Add information or hypothesis about sources of exposure to Pb and other metals

Response: Description of the environmental questionnaire and discussion of possible sources fo Pb and other metal exposure was in a previous publication (1). However, I have added a small amount of discussion of this issue.

Updated Manuscript Text: Page 16, Lines 297-303 “It is clear that more work to characterize sources of Pb and other heavy metal exposures in this environment is needed. An environmental health questionnaire in this population found no significant association between traditionally recognized heavy metal exposure risk factors in children (parent occupation, painted walls), and blood metal levels (1). One study from another section of Kampala found increased blood lead level in children with increased proximity to a local landfill (5).”

Compare the levels of metals in blood in study children and reference values in the general population. From this it should be clear that some issue on Zn in blood is present;

Response: It was the purpose of a previous publication to discuss the values in detail(1). Also, because our analysis of all metals was in whole blood, we are unable to comment on either Zn or Se deficiency. However, we do compare our results with a publication that similarly analyzed heavy metals in whole blood of children age 6-59 months from Kinshasa DRC.

Updated manuscript text: Page 15, Lines 274-279 “Although it is reasonable to suspect a high prevalence of Zn deficiency in our study population based on local diet and previous studies in similar populations (6-8), whole blood Zn cannot be used to diagnose deficiency so we were unable to determine rates of Zn deficiency in this study. The whole blood Zn levels in our study (median = 3.53 mg/L) were comparable but slightly lower than a population of the same age in Kinshasa, DRC (median = 5.0 mg/L) (9). “

Add info about the derivation of HAZ and its limitation when applied in Ugandan children;

Updated manuscript text: Page 16, Lines 311-314 “We used Epi info version 3.5.1 to derive HAZ scores, which is based upon a sample of formula-fed babies in the USA in 1978, which could overestimate undernutrition in our sample since the rate of growth in formula-fed infants is greater than breast-fed in the first year of life.”

Add the limitations of your study; Consider to smooth the conclusion of the manuscript, to include the weaknesses of the study and the fact that only Pb seems to exert a negative influence on growth.

Response: The limitations section was updated and expanded. Page 16

References:

1.	Cusick SE, Jaramillo EG, Moody EC, Ssemata AS, Bitwayi D, Lund TC, et al. Assessment of blood levels of heavy metals including lead and manganese in healthy children living in the Katanga settlement of Kampala, Uganda. BMC Public Health. 2018;18(1):717.

2.	Carrico C, Gennings C, Wheeler DC, Factor-Litvak P. Characterization of Weighted Quantile Sum Regression for Highly Correlated Data in a Risk Analysis Setting. J Agric Biol Environ Stat. 2015;20(1):100-20.

3.	Curtin P, Kellogg J, Cech N, Gennings C. A random subset implementation of weighted quantile sum (WQSRS) regression for analysis of high-dimensional mixtures. Communications in Statistics - Simulation and Computation. 2019.

4.	Team CMA, Mazzella M, Sumner SJ, Gao S, Su L, Diao N, et al. Quantitative methods for metabolomic analyses evaluated in the Children's Health Exposure Analysis Resource (CHEAR). J Expo Sci Environ Epidemiol. 2020;30(1):16-27.

5.	Graber LK, Asher D, Anandaraja N, Bopp RF, Merrill K, Cullen MR, et al. Childhood lead exposure after the phaseout of leaded gasoline: an ecological study of school-age children in Kampala, Uganda. Environ Health Perspect. 2010;118(6):884-9.

6.	Walker BE, Kelleher J. Plasma whole blood and urine zinc in the assessment of zinc deficiency in the rat. J Nutr. 1978;108(10):1702-7.

7.	Ndeezi G, Tumwine JK, Bolann BJ, Ndugwa CM, Tylleskar T. Zinc status in HIV infected Ugandan children aged 1-5 years: a cross sectional baseline survey. BMC Pediatr. 2010;10:68.

8.	Bitarakwate E, Mworozi E, Kekitiinwa A. Serum zinc status of children with persistent diarrhoea admitted to the diarrhoea management unit of Mulago Hospital, Uganda. Afr Health Sci. 2003;3(2):54-60.

9.	Tuakuila J, Kabamba M, Mata H, Mata G. Toxic and essential elements in children's blood (<6 years) from Kinshasa, DRC (the Democratic Republic of Congo). J Trace Elem Med Biol. 2014;28(1):45-9.
