# Supplementary material for: Environmental exposure to metal mixtures and linear growth in healthy Ugandan children
Source: PLoS One. 2020 May 15;15(5):e0233108. doi: 10.1371/journal.pone.0233108 (PMC7228047; doi:10.1371/journal.pone.0233108)
Supplement: Attachment — Submitted filename: Response letter 2nd revision.docx [file pone.0233108.s007.docx]

Dear Dr. Jose Moran and Reviewers,

Thank you for the time you have put into reviewing this paper. I appreciate the comments and have done my best to fully and thoughtfully respond to each. I hope that you find the responses have strengthened the manuscript as I certainly do. I look forward to any further communication.

In the following, please find the numbered comments from the reviewers followed by bulleted responses.

Sincerely,

Emily Moody

Reviewer #1:

1. Table 2 of this version needed still to improve. I saw the authors said adding a column of p-values to show the test (Wilcoxon rank sum test) of stunted children vs non-stunted children. However, I did not read any of this information in the table 2 nor in the text. The readers will not understand what for these p-values were.

- In response to the sum of comments on Table 2, and in an effort to make the table easy to read, I have decided to use the log2 transformed metals measures and to provide the mean (SD) rather than both mean (SD) and median (IQR). The t-test to compare stunted and non-stunted groups was done and is described in the methods section as well as the caption for the table.
- The original table with unadjusted metals measures, including both mean (SD) and median (IQR), and including stunted and non-stunted groups and the appropriate Wilcoxon rank sum test is now included in the supplemental material.
- METHODS - LINE 144-146: “Heavy metals data were log2 transformed because of right-skewness for comparability. We tested for differences in the transformed heavy metal concentrations in the stunted and non-stunted children using a t-test.”
- RESULTS – LINE 199-202: “Transformed heavy metal exposures are shown in Table 2; there were no significant differences in heavy metal exposures between children with stunting and with no stunting. Untransformed heavy metal exposures (S1 Table) and correlations between heavy metal exposures are included in supplemental materials (S1Fig).”
- CAPTION – LINE 205-208: “Heavy metal exposures for the study population (n=97), and for the stunted (n=22), and not stunted (n=75) stratified groups. Heavy metals data were log2 transformed because of right-skewness. The reported p-value is for a t-test comparing heavy metals exposures in stunted and not stunted populations. All p-values are not significant.”

2. I still suggest that sepreate into 2 groups (stunted children vs non-stunted children) of the metal data distributions for the table 2, as I suggested previously.
   - Thank you for the suggestion, I’m sorry I did not clearly understand your previous request. This has been included.

Reviewer #2: The manuscript has been revised and improved, but still some points rise concern and need interventions. Particularly:

1. the authors state that they measured heavy metals. Unfortunately this is questionable, as
   • As is a semi metallic element,
   • Se in a non metallic element
   Sorry for not having pointed this before. Please revise the text considering this issue.

- Thank you for the comment. I have included a brief definition and citation in the introduction for readers who may be less familiar.
- LINE 74-76: “Heavy metals are a diverse group of naturally occurring metallic elements with high density and toxicity to humans; some are also essential nutrients and are referred to as essential heavy metals (1).”
- I have updated the title and text to more clearly say “heavy metal” rather than “metal” for greater accuracy.

2. Line 42 in abstract. The negative effect is presumed as well.
   - This is true. Text was updated to read:
   - LINE 36-38 “The WQS models separated nutritional metals with presumed positive effect on HAZ (Se and Zn) from toxic metals with a presumed negative effect on HAZ (Pb, As, Cd).”
3. Discussion: I still think that the results of this study do not demonstrate, but suggest that children are experiencing detrimental effects associated with exposure to environmental pollutants. This comment was previously given, but authors did not take into account it. Moreover, the effect seems to be associated with Pb and not with other pollutants.
   - I agree with your observation and interpretation. I have revised in an attempt to change all similar language to more appropriately express that.
   - LINE 44-47 “These findings suggest that healthy children in this neighborhood of Kampala, Uganda, who have a high burden of toxic heavy metal exposure, may experience detrimental health effects associated with these exposures in an environment where exposure sources are not well characterized.”
   - LINE 253-254: “These findings suggest that elevated Pb and lower Se in young children in the Katanga region of Kampala, Uganda are associated with decreased HAZ.”
4. Paragraph Linear Growth. Please add here the information about the HAZ score. That is:
   1-this was developed for US children fed with formula.
   2. this may be not suitable for breast-feed children up to 1 year. Please specify where we can find the algorithm and apply it. Add references too.
   - Thank you for attending to this important detail. The following text was changed:
   - LINE 131-134: “Height-for-age Z-score (HAZ) was calculated for each child using Epi Info version 3.5.1 from Centers for Disease Control and Prevention, which applies height and weight measurements against the Centers for Disease Control/World Health Organization 1978 growth references (2, 3).”
   - LINE 329-337 “We used Epi info version 3.5.1 to derive HAZ scores, which uses growth charts developed by the US CDC/WHO in 1978 based upon a US population of formula-fed infants (2, 4). Use of this reference for our population of primarily breast fed babies in Uganda could result in overestimating undernutrition in the babies under 12 months since the rate of growth in formula-fed infants is generally greater than breast-fed infants in the first year of life. We don’t expect this significantly affected our results because of the 19 infants under 12 months of age in our sample, there were only 2 with HAZ <-2. The proportion of infants under 12 months with stunting (10.5%) was less than in the over 12-month old group (25.6%) and the overall sample (22.7%).”
5. Given that the HAZ index in not working very well for children younger than 1 y, could you please state how many of the stunting children were in this range of age?
   - Of course. There were 19 children younger than 1 year, two (11%) of whom were stunted. Of the 78 children older than one year, 20 (26%) were stunted.
   - LINES 331-337 “We don’t expect this significantly affected our results because of the 19 infants under 12 months of age in our sample, there were only 2 with HAZ <-2. The proportion of infants under 12 months with stunting (10.5%) was less than in the over 12-month old group (25.6%) and the overall sample (22.7%).”
6. Table 1: divide the table and compare the groups of stunting and non-stunting children.
   - The table has been updated. A chi-square test compared the stunted and non-stunted groups for dichotomous population characteristic variables (sex, mother’s education, and whether the child had been admitted to the hospital). The Mann-Whitney U test was used for continuous variables (sex and HAZ). This p-value is reported with clear explanation in the methods section and in the table caption.
   - LINE 139-143: “Initial descriptive statistics were for the whole study population and groups stratified by stunting status. A chi-square test compared the stunted and non-stunted groups for dichotomous population characteristic variables (sex, mother’s education, and whether the child had been admitted to the hospital). The Mann Whitney U test was used for continuous variables (sex and HAZ).”
   - CAPTION – LINE 193-196: “Study population characteristics for the entire study group and groups stratified by stunting status. Reported p-value for Chi-Square test for dichotomous variables (sex, mother’s education, and whether the child had ever been admitted to the hospital), and for Mann-Whitney U test for continuous variables (age and HAZ).”

7. Table 2 and t-test between stunted and non stunted children. Please, divide children based on their classification of stunting /non-stunting and compare the groups. IN the present version, a p value is given, but it is not clear why. Specify it in the legend.

- In response to the sum of comments on Table 2, and in an effort to make the table easy to read, I have decided to use the log2 transformed metals measures and to provide the mean (SD) rather than both mean (SD) and median (IQR). The t-test to compare stunted and non-stunted groups was done and is described in the methods section as well as the caption for the table.
- The original table with unadjusted metals measures, including both mean (SD) and median (IQR), and including stunted and non-stunted groups and the appropriate Wilcoxon rank sum test is now included in the supplemental material.
- METHODS - LINE 144-146: “Heavy metals data were log2 transformed because of right-skewness for comparability. We tested for differences in the transformed heavy metal concentrations in the stunted and non-stunted children using a t-test.”
- RESULTS – LINE 199-202: “Transformed heavy metal exposures are shown in Table 2; there were no significant differences in heavy metal exposures between children with stunting and with no stunting. Untransformed heavy metal exposures (S1 Table) and correlations between heavy metal exposures are included in supplemental materials (S1Fig).”
- CAPTION – LINE 205-208: “Heavy metal exposures for the study population (n=97), and for the stunted (n=22), and not stunted (n=75) stratified groups. Heavy metals data were log2 transformed because of right-skewness. The reported p-value is for a t-test comparing heavy metals exposures in stunted and not stunted populations. All p-values are not significant.”

8. The correlation between metals is not relevant to the study; I propose to remove Figure 1, especially considering that the data on metals were published before.
   - The correlation plot has been moved to the supplemental materials where a reader may find the information if they are interested.
9. Discussion.
   The study does not demonstrate but suggest, a role of lead on stunting in Ugandan children; this limit the generalization to the other investigated metals.
   Moreover, it should be clarified how many children, classified as stunting, were below 12 months, considering the limitation of the HAZ index for those children.
   In general, author should add some additional info in the manuscript, and use more caution in the interpretation of their results.

- Thank you for your contribution to this manuscript. These suggestions have been taken into account and answered in the above numerated items.

References:

1.	Tchounwou PB, Yedjou CG, Patlolla AK, Sutton DJ. Heavy metal toxicity and the environment. Exp Suppl. 2012;101:133-64.

2.	Center for Disease Control. Use of World Health Organization and CDC Growth Charts for Children Aged 0–59 Months in the United States. September 10, 2010.

3.	U.S. Department of Health & Human Services. Epi Info: Center for Surveillance, Epidemiology & Laboratory Services; [Available from: <https://www.cdc.gov/epiinfo/index.html>.

4.	Dibley MJ, Goldsby JB, Staehling NW, Trowbridge FL. Development of normalized curves for the international growth reference: historical and technical considerations. Am J Clin Nutr. 1987;46(5):736-48.
