# Supplementary material for: Environmental exposure to metal mixtures and linear growth in healthy Ugandan children
Source: PLoS One. 2020 May 15;15(5):e0233108. doi: 10.1371/journal.pone.0233108 (PMC7228047; doi:10.1371/journal.pone.0233108)
Supplement: Attachment — Submitted filename: Dear Editor.docx [file pone.0233108.s008.docx]

April 23, 2020

Dear Editor Jose Moran and Reviewers,

I hope you are all well during this difficult time. I appreciate your continued work to improve our manuscript and feel that it has benefitted significantly from your contributions. I have thought carefully about your comments and responded as best I could. I hope you find the updated table and nomenclature acceptable for publication. As with the previous revision, I have included a document with all of the de-identified data to make the data underlying the published findings publicly available. I eagerly await to hear of next steps.

Sincerely,

Emily Moody

COMMENTS:

Reviewer #1: The new table 2 used a log2 tranformation of metal concentration, but the first column used uints without transformed, such as Pb(ug/dL), Al(ug/L), Cd(ug/L), Se(ug/dL), Zn(mg/L), were incorrected. That is why the negative numbers appears. Think about that, -2.15 ug/L As in blood, is so weird. If the authors used log2 transformation, then the units (ug/dL, ug/L, mg/L) in the table 2 should be ommitted. Otherwise, please used geometric means.

Thank you for this comment. I agree that units should have been omitted from that table and that using transformed data in this table is confusing. The table now includes the median and IQR for the entire cohort, and stunted and non-stunted subgroups and a Wilcoxan Rank Sum test for the test for differences in distribution. The mean and sd are included in the supplemental table for anyone interested in this information.

Reviewer #2: The authors addressed the comments and the manuscript is improved.
Still there are two suggestions:

1- Heavy metals. I previously commented that the trace elements measured by this research are not all "heavy metals", but there are semi-metallic elements and non-metallic elements. It is unclear why, following this comment, the authors transformed all the noun "metals" in the manuscript as "heavy metals". Please consider revising this.

I appreciate this comment and have thought about this a lot. I struggled to find a noun that can simply and accurately describe the mixtures. The common usage in environmental chemistry and environmental health includes As and Se as “heavy metals” although this may be an antiquated and ultimately inaccurate term. I have removed the use of “heavy metal” and have continued to generally refer to the mixture as metals with the following explanation. Where possible, I have used other descriptors.
“For simplicity, we refer to our mixture including lead (Pb), arsenic (As), cadmium (Cd), selenium (Se), and zinc (Zn), as metals, although Zn is a semi-metallic element and Se is a non-metallic element.”

2- Table 2 and log transformed data. Log transformation is a very common way to obtain normal distribution of data, useful for applying parametric statistics. This was properly done and I appreciate it. However, when reporting data in tables they should be reported as normal values. To report data in the log transformed form is not very clear for readers and should be avoided.

As in the comment above, I agree that this was not the correct choice. I have changed the table so that it presents the median and IQR for the entire cohort, and stunted and non-stunted sub groups and a Wilcoxan Rank Sum test for the test for differences in distribution. The mean and sd are included in the supplemental table.
